# A new approach to evaluating loop inconsistency in network meta-analysis

**DOI:** 10.1002/sim.9872

**Published:** 2023-09-28

**Authors:** Rebecca M. Turner, Tim Band, Tim P. Morris, David J. Fisher, Julian P. T. Higgins, James R. Carpenter, Ian R. White

**Affiliations:** 1 MRC Clinical Trials Unit at University College London, London, UK; 2 Advanced Research Computing, University College London (UCL), London, UK; 3 Population Health Sciences, Bristol Medical School, University of Bristol, Bristol, UK; 4 Department of Medical Statistics, London School of Hygiene and Tropical Medicine, London, UK

**Keywords:** global test, inconsistency, loop inconsistency, network meta-analysis

## Abstract

In network meta-analysis, studies evaluating multiple treatment comparisons are modeled simultaneously, and estimation is informed by a combination of direct and indirect evidence. Network meta-analysis relies on an assumption of consistency, meaning that direct and indirect evidence should agree for each treatment comparison. Here we propose new local and global tests for inconsistency and demonstrate their application to three example networks. Because inconsistency is a property of a loop of treatments in the network meta-analysis, we locate the local test in a loop. We define a model with one inconsistency parameter that can be interpreted as loop inconsistency. The model builds on the existing ideas of *node-splitting* and *side-splitting* in network meta-analysis. To provide a global test for inconsistency, we extend the model across multiple independent loops with one degree of freedom per loop. We develop a new algorithm for identifying independent loops within a network meta-analysis. Our proposed models handle treatments symmetrically, locate inconsistency in loops rather than in nodes or treatment comparisons, and are invariant to choice of reference treatment, making the results less dependent on model parameterization. For testing global inconsistency in network meta-analysis, our global model uses fewer degrees of freedom than the existing design-by-treatment interaction approach and has the potential to increase power. To illustrate our methods, we fit the models to three network meta-analyses varying in size and complexity. Local and global tests for inconsistency are performed and we demonstrate that the global model is invariant to choice of independent loops.

## 1 Introduction

Network meta-analysis is an important and popular way to compare a number of treatments for a given condition (1). In a network meta-analysis, studies evaluating multiple treatment comparisons are modeled simultaneously, and estimation is based on a combination of direct and indirect evidence (2–6). Direct evidence on treatments A vs B is obtained from a study comparing treatments A and B, while indirect evidence is obtained through a common comparator, for example, from studies comparing A vs C and studies comparing B vs C. Network meta-analysis rests on an assumption of consistency, meaning that direct and indirect evidence should agree for each comparison (6). An important part of performing a network meta-analysis is therefore to test for inconsistency.

Tests for inconsistency may be local or global. Local tests commonly use node-splitting: a model is fitted in which a chosen treatment effect is allowed to differ between studies that directly estimate the effect and other studies that contribute only indirect evidence (7).

Global tests attempt to summarize evidence for inconsistency across the entire network and can be used to indicate whether local tests should be performed. Lu & Ades argued that the number of degrees of freedom for inconsistency in a network equals the number of independent loops and proposed a corresponding model (8). Higgins et al showed that this is true when all studies are two-arm studies but not true when the network includes multi-arm studies (9). In the multi-arm study case, loop inconsistency is hard to define because different ways of parameterizing the multi-arm trials can result in varying numbers of loops in the network. Higgins et al defined inconsistency as design-by-treatment interaction (DBTI), where design refers to the set of treatments compared within the trial, and proposed fitting a model allowing for all design-by-treatment interactions (9). However, the number of degrees of freedom for inconsistency in a DBTI model can be much larger than the number of loops, with consequent loss of power for testing inconsistency and practical implications.

Here we propose new local and corresponding global tests for inconsistency. Because inconsistency is a property of a loop, we locate the local test in a loop, and define a model with one inconsistency parameter per loop, which can be interpreted as loop inconsistency. To give a global test for inconsistency, the model can be extended across multiple independent loops. This means the new model uses one degree of freedom per loop. The model builds on the idea of *node-splitting* or *side-splitting* as introduced by Dias et al (7) and modified by White (10). Most existing models for inconsistency handle treatments asymmetrically, which introduces an unnecessary arbitrary choice into the analysis.

We show that our new proposed models handle treatments symmetrically, are invariant to the choice of reference treatment, allow for multi-arm trials and are more careful about spending degrees of freedom. We provide an algorithm for identifying independent loops within a network and show that the global model is invariant to choice of loops.

In Section 2, we describe our proposed model for splitting a single loop, first in a simple network and then in a general network, and then present our global model for splitting multiple loops and propose how to choose a set of independent loops to be split simultaneously. In Section 3, our new methods are demonstrated through application to three example networks varying in size and complexity. We conclude with a discussion in Section 4.

## 2 Methods

We assume random effects models for pairwise comparisons of multiple treatments, as proposed first by Higgins and Whitehead (2), and later extended by Lu and Ades (4). The generic model described below is written as a “contrast-based” model and follows the notation used by White et al. (11).

Suppose the network includes treatments *A*, *B*, *C* …, where (without loss of generality) treatment *A* is regarded as the overall reference treatment. Initially, we assume that treatment *A* is included in every study. Let *d* = 1, …, *D* index the designs included in the network, where design refers to the set of treatments compared in the trial. Let $y_{di}^{AJ}$ be the observed treatment contrast of a generic treatment J with treatment *A* in study *i* with design *d*, where $y_{di}^{AJ}$ may represent any effect measure such as a mean difference, standardised mean difference, log odds ratio or log risk ratio. We assume the following model for the observed data:

(1) $$y_{di}^{AJ}=\delta^{AJ}+\beta_{di}^{AJ}+\omega_{d}^{AJ}+\varepsilon_{di}^{AJ},J=B,C,\ldots$$

where *δ^AJ^* represents the average contrast between treatments J and A, $\beta_{di}^{AJ}$ allows for between-study heterogeneity, $\omega_{d}^{AJ}$ represents an inconsistency term and $\varepsilon_{di}^{AJ}$ represents within-study sampling error. The $\beta_{di}^{AJ}$ are treated as correlated random effects:

$$\beta_{di}∼N(0,\Sigma ).$$

In this article, we make the simplifying assumption that between-study heterogeneity variances are equal across all pairwise comparisons in the network. Under this assumption, the between-study covariance matrix **Σ** has diagonal entries *τ*^2^ and off-diagonal entries *τ*^2^/2. However, this assumption is not required for implementation of the loop-splitting methodology described below.

We note that model (1) above is a generic model and that additional assumptions are required for the inconsistency terms $\omega_{d}^{AJ}$. These assumptions determine whether the model is a consistency model or an inconsistency model and, if the latter, what form of inconsistency is modeled. We create a consistency model by setting $\omega_{d}^{AJ}$ to 0 for all *d*, *J*. Under an inconsistency model, allowance is made for disagreement between designs by including non-zero $\omega_{d}^{AJ}$. In this article, we treat the $\omega_{d}^{AJ}$ as fixed effects throughout. Below we consider several different inconsistency models.

For the general case in which no treatment is common to all studies, we assign a study-specific reference treatment A_*i*_ in each study *i*, and define all treatment contrasts above with respect to A_*i*_ rather than A, using a model derived from the model above.

### 2.1 Loop splitting in simple networks

**Figure 1:**
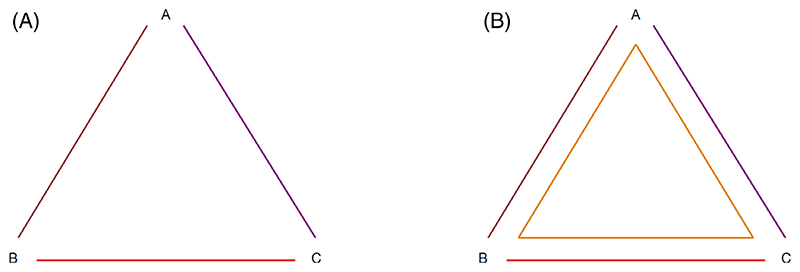
A simple network AB/AC/BC and a larger network AB/AC/BC/ABC. Each line or shape represents one or more trials comparing all joined treatments.

We first consider a simple network AB/AC/BC (Figure 1A) and a larger network AB/AC/BC/ABC (Figure 1B). We propose an inconsistency model that handles treatments symmetrically and is invariant to choice of reference treatment, and show how inconsistency terms are assigned under this model in comparison with previous models (7,8,10). We consider five different models for both networks (Table 1): a consistency model; the asymmetrical (7) and symmetrical (10) models for splitting side B-C; the Lu-Ades inconsistency model with reference A (8); and our proposed inconsistency model for the directed loop *A* → *B* → *C* → *A*. Table 1 shows the true mean treatment effect(s) in each trial design; observed treatment effects vary around these due to between-study heterogeneity and within-study variation.

In the simple network excluding the ABC design, it makes no difference where the inconsistency term is assigned: to the C vs B contrast or equally to all contrasts. These approaches result in the same model; there is nothing in the data to distinguish them.

In the larger network including the ABC design, it does matter where we add the inconsistency term, because there is information in the data to distinguish between the models. The asymmetrical side-splitting model allows the C vs A contrast to differ between the two-arm (AC) and three-arm (ABC) studies, but does not allow the other contrasts to differ between the two-arm and three-arm studies. The symmetrical side-splitting model allows the B vs A and C vs A contrasts (but not the C vs B contrast) to differ between the two-arm and three-arm studies, and forces these differences to be equal and opposite. The Lu-Ades inconsistency model allows the C vs B contrast to differ between the two-arm (BC) and three-arm (ABC) studies, but does not allow the other contrasts to differ between the two-arm and three-arm studies. Our new proposed model is the only model which handles the treatments symmetrically: it adds equal amounts of inconsistency to each two-arm study. The direction of inconsistency matches the direction of the loop, so for example, the C vs A contrast has a negative term because *A → C* goes against the loop direction. We note that choosing the opposite direction for the loop would result in the same model but with a reversed sign for the inconsistency term.

### 2.2 I Loop splitting in general networks

We extend this idea for a three-treatment loop in a general network with reference treatment *A*. Consider the loop *B → C → D → B*. (Excluding the reference treatment *A* from the loop makes the description easier.) We introduce inconsistency terms to studies that contain exactly two of the treatments in the loop:

- For studies containing *B* and *C* (but not *D*), the inconsistency terms are those from splitting *B*-*C*, with parameter *ω*/3.
- For studies containing *C* and *D* (but not *B*), the inconsistency terms are those from splitting *C-D*, with the same parameter *ω*/3.
- For studies containing *B* and *D* (but not *C*), the inconsistency terms are those from splitting *D*-*B* with the same parameter *ω*/3. Note that the direction *D*-*B* is used rather than *B*-*D*, so that inconsistency accumulates round the loop.
- For studies containing *B*, *C* and *D*, no inconsistency terms are added.

The symmetrical approach to side-splitting is used (10). Implementation is via network meta-regression on three covariates defined in Table 2a, all with the same coefficient *ω*/3 (noting that dividing by 3 ensures that inconsistency sums to *ω* round the 3-treatment loop). This defines one new term for the model. Splitting different loops generates different terms and we will discuss how to split multiple loops simultaneously in the next section. After estimating the coefficient *ω*/3 within a network meta-regression model, a test for inconsistency within the three-treatment loop is performed using a Wald test with test statistic $(\hat{\omega }/SE(\hat{\omega }){)}^{2}$, which is referred to a $\chi_{1}^{2}$ distribution. We note that allowance is made for uncertainty in the estimated between-study variance.

We now extend this to a four-treatment loop *B → C → D → E → B*, again in a general network with reference treatment *A*. We introduce inconsistency terms to studies that contain two or three of the treatments in the loop. For studies containing two treatments that are adjacent in the loop, the inconsistency terms are assigned in the same way as in the three-treatment loop. For example, for studies containing *B* and *C* (but not *D* or *E*), the inconsistency terms are those from splitting *B*-*C*, with parameter *ω*/4 (where dividing by 4 again ensures that inconsistency sums to *ω* round the loop). For studies containing two treatments that are not adjacent in the loop, *B* and *D* for example (but not *C* or *E*), no inconsistency term is required. For studies containing three of the four treatments in the loop, the inconsistency terms are assigned to the two treatment comparisons in the study which coincide with edges of the loop. For example, for studies containing *B*, *C* and *D* (but not *E*), the inconsistency terms are those from splitting *B*-*C* and *C*-*D*. For studies containing three of the treatments in the loop, we assign inconsistency terms of *ω*/8 rather than *ω*/4 to each edge. We justify this approach later by demonstrating equivalence between global models splitting three-treatment loops (in which each study contains at most two of the treatments in the loop) and models splitting three-treatment and four-treatment loops (in which some studies contain three of the treatments in the loop). For studies containing *B*, *C*, *D*, and *E*, no inconsistency terms are added.

The inconsistency terms assigned to studies of different designs when splitting a four-treatment loop are presented in Table 3. Implementation of the model splitting a four-treatment loop is via network meta-regression on four covariates defined in Table 2b, all with the same coefficient *ω*/4, so that inconsistency accumulates to *ω* round the loop. After estimating the coefficient *ω*/4 within a network meta-regression model, a test for inconsistency is performed using a Wald test with test statistic $(\hat{\omega }/SE(\hat{\omega }){)}^{2}$, which is referred to a $\chi_{1}^{2}$ distribution. Covariates for implementation of a model splitting a five-treatment loop are defined in Table S1. The loops considered above exclude the network reference treatment, *A*. For loops including the reference treatment, the coding of the network meta-regression covariates differs; examples are provided in Tables S2 and S3.

### 2.3 Splitting multiple loops

In this section, we propose a global loop-inconsistency model for networks containing multiple loops. In a network including only pairwise trials, let *n* represent the number of treatment comparisons informed by data and let *k* represent the number of treatments in the network. Lu and Ades showed that the number of independent loop inconsistencies in the network is equal to *n* − *k* + 1 (8). Before we consider networks including multi-arm trials, we first set out an explanation for the number of independent loops in a network of pairwise trials or a network in which all comparisons present in multi-arm trials are also present in one or more pairwise trials.

It is helpful to consider the graphical representation of the network, following Lu and Ades (8), and call this graph *𝒢* = (***𝒯*, *𝓔***), where ***𝒯*** is the set of *k* nodes representing treatments and *ω* is the set of *n* undirected edges representing treatment comparisons informed by data. A global loop-inconsistency model must include one basic or functional parameter for every comparison informed by data and therefore includes *n* parameters. A spanning tree for graph *𝒢* includes the minimal number of *k* − 1 edges required to span the network; the dimension of the spanning tree is equal to the number of basic parameters in the model. Loops in the graph *𝒢* are created when we add in additional edges beyond the number of edges included in a spanning tree. The number of independent loops in the graph is therefore equal to the difference between the number of parameters included in the model and the dimension of the spanning tree, that is, *n* − *k* + 1. Each loop represents a potential source of inconsistency and we will include one inconsistency parameter for every independent loop.

**Figure 2:**
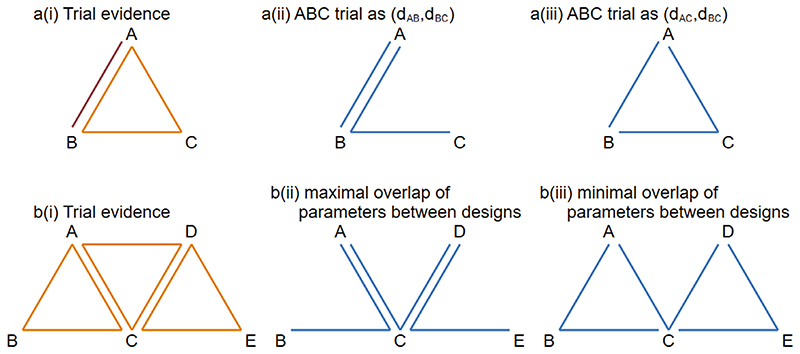
(A) Network including AB and ABC trials: (i) trial evidence; (ii) ABC trial parameterized as (d_AB_, d_BC_); (iii) ABC trial parameterized as (d_AC_, d_BC_). (B) Network including ABC, ACD, and CDE trials: (i) trial evidence; (ii) parameterization with maximal overlap of parameters between designs; (ii) parameterization with minimal overlap of parameters between designs. Red/orange lines represent trial evidence, while blue lines represent model parameterizations.

We now consider networks including multi-arm trials, in which one or more comparisons present in multi-arm trials are not present in any pairwise trials. In such a network, there are different options for how to parameterize a global loop-inconsistency model (8,12). We consider models that represent the parameterization in a natural way; different parameterizations can produce different numbers of independent loops. To illustrate this, we consider a small network including *AB* trials and *ABC* trials (Figure 2A[i]). We can choose whether to parameterize the *ABC* trial as (*d_AB_*, *d_AC_*), (*d_AB_*, *d_BC_*) or (*d_AC_*, *d_BC_*). Choosing (*d_AB_*, *d_BC_*) would mean that no loops are created in the inconsistency model, as illustrated in Figure 2A(ii). However, choosing *(d_AC_, d_BC_)* would mean that one loop is created, as illustrated in Figure 2A(iii). The difference between the minimal and maximal numbers of loops can be greater in networks including more multi-arm trial designs. For example, in Figure 2B we consider a network including *ABC*, *BCD*, and *CDE* trials. If we choose a parameterisation which maximises overlap of parameters between the designs, no loops are created in the model (Figure 2B[ii]). Alternatively, choosing a parameterisation with minimal overlap of parameters between designs would produce two loops (Figure 2B[iii]).

Choice of parameterization has implications for how much variation among trials is modeled as between-trial heterogeneity and how much is modeled as inconsistency. This choice cannot be informed by the data. Previous authors have chosen to parameterize inconsistency models in a way that maximizes the number of loops (8,12). In this article, we will take a different view and choose parameterizations that minimize the number of loops, for the following reason. Minimizing the number of inconsistency parameters in the model means that variation among trials is modeled as heterogeneity wherever possible and only modeled as inconsistency where necessary. Since between-trial heterogeneity is usually assumed equal across comparisons, modeling variation as heterogeneity wherever possible will result in a model including fewer parameters in total, with a simpler interpretation and representing a more coherent extension from pairwise meta-analysis. For example, the parameterization in Figure 2A(ii) requires a heterogeneity parameter to model the between-trial variation on comparison *A* vs *B*, but does not require an inconsistency parameter. Similarly, the parameterisation in Figure 2B(ii) requires heterogeneity parameters to model the between-trial variation on comparisons *A* vs *C* and *C* vs *D*, but requires no inconsistency parameters. Conversely, the parameterisations in Figure 2A(iii) and B(iii) require inconsistency parameters but no heterogeneity parameters.

We now consider the graphical representation *𝒢* = (***𝒯*, *𝓔***) of a generic network including multi-arm trials, where *𝒯* is the set of *k* nodes representing treatments and *ω* is the set of undirected edges representing treatment comparisons informed by data. Choosing a parameterisation for the global loop-inconsistency model is equivalent to choosing a union of spanning trees for every trial design in the network. We use *𝒢_p_* = (***𝒯***, *𝓔_p_*) to denote a graph corresponding to parameterisation *p*, where *ω_p_* is the set of *n_p_* undirected edges included in the chosen union of spanning trees. As discussed previously for networks including only pairwise trials, loops in the graph *𝒢_p_* are created when we add in additional edges beyond the *k* − 1 edges included in a spanning tree. The number of independent loops in a graph representing parameterization *p* is therefore equal to *n_p_* − *k* + 1.

We have developed a new algorithm to identify a model parameterization that minimizes the number of loops, the code is provided in supplementary material. Briefly, a pair of recursive functions are used to create an array of network adjacency matrices representing all possible unions of spanning trees for the set of trial designs in the network. An adjacency matrix including the fewest edges is chosen (arbitrarily, if more than one have the fewest edges) as the minimal network spanning matrix; because this includes the minimum number of additional edges beyond *k* − 1, it will produce the smallest number of loops. Next, we identify the loops present in the minimal network spanning matrix. The edges present in the matrix are used to construct a spanning tree: one treatment is chosen arbitrarily as the root of the tree, and edges are added to the tree one by one if they are connected to the existing tree by one treatment vertex. Any remaining edges (not yet added) have both treatments already included in the spanning tree; each of these creates a loop that is identified by finding the lowest common ancestor of the two treatment vertices. We note that the set of independent loops present is not necessarily unique. However, in later sections we shall demonstrate that sets of independent loops are equivalent to each other.

After identifying a set of independent loops, a global loop-splitting model is implemented via network meta-regression. The covariates in the meta-regression are defined as described above for models splitting a single loop, but we now include one regression coefficient for each loop to be split. For example, to split two three-treatment loops *ABC* and *BCD* simultaneously, we would include coefficients *ω_ABC_*/3 and *ω_BCD_*/3. After fitting a network meta-regression model, a test for global inconsistency is performed using a global Wald test with test statistic ${\hat{\omega }}^{\prime }V^{-1}\hat{\omega }$, where $\hat{\omega }$ is a vector of estimated inconsistency parameters and ***V*** is its estimated variance. The test statistic is referred to a $\chi_{k}^{2}$ distribution, where *k* is the number of loops being split simultaneously.

### 2.4 Graphical methods for visualizing inconsistency

We have explored how to visualize the estimated inconsistencies within multiple loops in a network, to aid interpretation of the results from a global inconsistency model. To illustrate where there is evidence of inconsistency across the network, we propose plotting the set of independent loops fitted in a global model, using colors that represent the degree of significance (*P*-value) of the corresponding local inconsistency estimate. This will help meta-analysts identify which treatment loops should be investigated for potential causes of inconsistency (6). Because the global model does not in general correspond to a unique set of loops, plotting just one such set may lead to overinterpretation of the loops that contribute least or most inconsistency to the network. We therefore propose multiple plots, each plotting a separate set of loops.

## 3 Application To Example Network Meta-Analyses

### 3.1 Smoking cessation network

**Figure 3:**
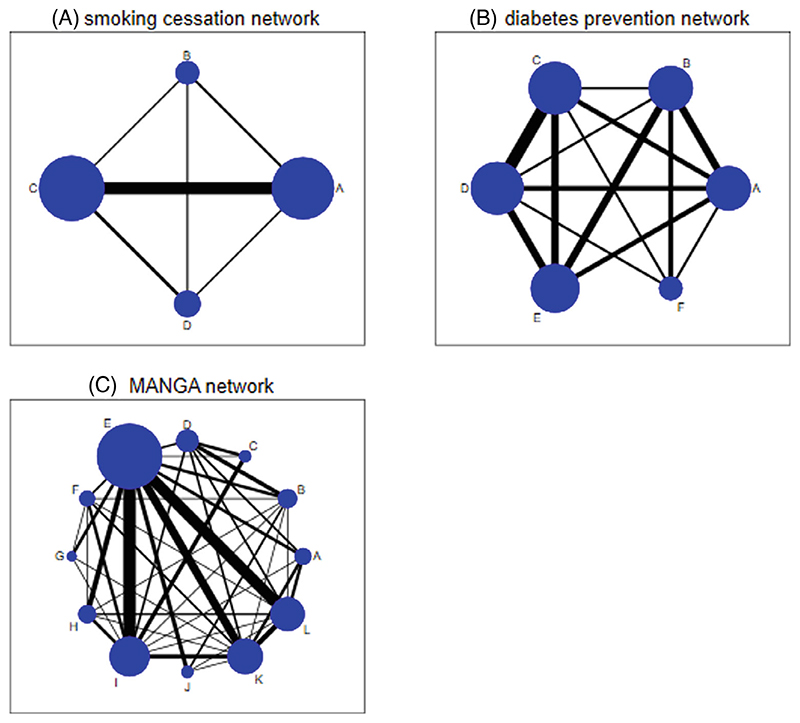
Example network meta-analyses: (A) smoking cessation network; (B) diabetes prevention network; (C) MANGA network.

Local and global tests for inconsistency are first applied to a commonly analyzed network meta-analysis data set including only four treatments (8). The network includes 24 trials comparing treatments for smoking cessation counseling: no intervention (*A*); self-help (*B*); individual counselling (*C*) and group counseling (*D*) (Figure 3A) 13). The outcome measured is successful cessation of smoking at 6-12 months. Direct evidence is available on all six pairwise comparisons *AB* (3 trials), *AC* (15 trials), *AD* (2 trials), *BC* (2 trials), *BD* (2 trials), and *CD* (4 trials) and there are two three-arm trials with designs *ACD* and *BCD*.

In the smoking cessation network, all comparisons present in multi-arm trials are also present in one or more pairwise trials. This means that the global inconsistency model includes a fixed number of independent loops, equal to 6 − 4 + 1 = 3. As discussed by Lu and Ades (8), there are seven different possible loops in the network, but only three of these are independent.

In Table 4, we present results from splitting each of the seven loops in the network: ABC, ABD, ACD, BCD, ABCD, ABDC, and ACBD. There is no evidence of inconsistency in any of the loops. We note that reversing the order of a loop gives the same results with a change of sign, as expected, for example, splitting ACB produces an inconsistency parameter estimate of 0.343 (0.745) (results not shown), where splitting ABC produces an inconsistency parameter of -0.343 (0.745). This makes no difference to the interpretation of the results. By applying our algorithm to identify a minimal network spanning matrix and then to identify loops present, we find that ABD, ACD, and BCD are independent loops. In a small network such as this, it is easy to see from Figure 1 that these three loops are independent. The results from fitting a global loop-inconsistency model to split loops ABD, ACD, and BCD are presented in Table 4. We find no global evidence of inconsistency across the network (*P* = .67).

The set of independent loops is not unique. By reordering the treatments and rerunning the algorithm we can identify a different set of independent loops, for example, ABD, BCD, and ABDC. Table 4 presents results from fitting the global loop-inconsistency model to three possible sets of independent loops. Jointly splitting any three independent loops produces the same model and we see that a test for global inconsistency produces identical results in each case, as expected. In principle there is no reason to present results from splitting more than one set of independent loops, but in practice this is useful to avoid overinterpretation of where inconsistency lies.

**Figure 4:**
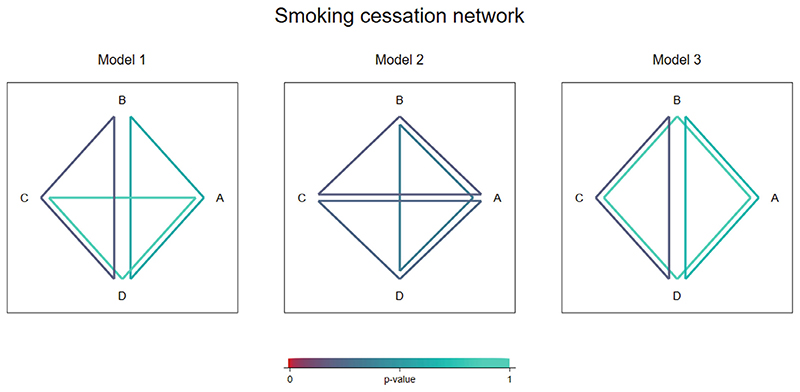
Inconsistency visualization plots illustrating results from three different global inconsistency models fitted to the smoking cessation network.

Figure 4 illustrates the results from the three different global models presented in Table 4. In the first and third models, we see that the strongest evidence for inconsistency lies within the BCD loop, while the *P*-value is much higher in the other two loops. However, there is no significant evidence for inconsistency in any of the loops. In the second model, the *P*-values associated with each loop are very similar and there is again no evidence for inconsistency.

### 3.2 Diabetes prevention network

As a second example, we test for inconsistency in a network meta-analysis comparing the effectiveness of antihypertensive drugs for prevention of diabetes (14). The network includes 22 trials comparing 6 different treatments: diuretic (A), placebo (B), beta blockers (C), calcium-channel blockers (D), angiotensin-converting-enzyme inhibitors (E), and angiotensin-receptor blockers (F) (Figure 3B). The primary outcome for efficacy was new cases of diabetes observed over the trial duration period. The network includes four three-arm trials with designs ABC, ADE, and CDE (2 trials) and direct evidence is available on all pairwise comparisons except EF.

Comparisons BC and DE are present in multi-arm trials but not in any pairwise trials, meaning that there are multiple ways to parameterize the global loop-inconsistency model. We apply our algorithm to identify a minimal network spanning matrix including 12 treatment comparisons and a set of 7 independent loops: ABD, ACD, ABE, ACE, ABF, ACF, and ADF. The results from fitting a global loop-inconsistency model to split these seven loops are presented in Table 5. There is no global evidence of inconsistency across the network (*P* = .48). As in the previous example, the results from jointly splitting different sets of independent loops are identical (Table 5).

**Figure 5:**
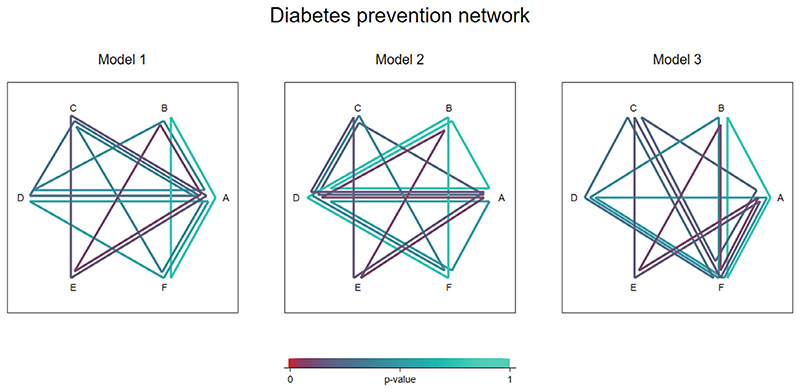
Inconsistency visualization plots illustrating results from three different global inconsistency models fitted to the diabetes prevention network.

Figure 5 illustrates the results from the three different global models. In the first model we see that the evidence for inconsistency appears strongest in the ABE loop. In the second model the strongest evidence appears to lie within the AEBD loop while in the third model it appears to lie within the AFBE loop. While the apparent sources of inconsistency depend on the set of loops split, the strongest evidence for inconsistency is found in the same part of the network under each model, and *P*-values from the corresponding local tests for inconsistency are identical to 2 decimal places (*P* = .11).

### 3.3 MANGA network

We now explore inconsistency in a much larger network meta-analysis comparing the efficacy and acceptability of 12 antidepressant drugs for treatment of adults with major depressive disorder (15). The primary outcome for efficacy was response rate at 8 weeks and the treatments compared were bupropion (A), citalopram (B), duloxetine (C), escitalopram (D), fluoxetine (E), fluvoxamine (F), milnacipran (G), mirtazapine (H), paroxetine (I), reboxetine (J), sertraline (K), and venlafaxine (L). The network includes 111 trials in total, of which two are three-arm trials with design EIK. All comparisons present in the multi-arm trials are also present in one or more pairwise trials, meaning that there is only one way to parameterize the global loop-inconsistency model. The number of different treatment comparisons evaluated in one or more trials is 42 and the number of independent loops is therefore equal to 42 − 12 + 1 = 31.

By applying our algorithm, we identify a set of 31 independent loops. Results from fitting a global loop-inconsistency model to split these loops simultaneously are presented in Table 6. We find no global evidence of inconsistency across the network (*P* = .51). In addition, we present identical results from splitting two different sets of independent loops, as in the previous two examples. We have not attempted to create visualization plots for loop inconsistencies across the MANGA network. The complexity of the network (Figure 3) means that many loops overlap and cross each other, and the inconsistency visualization plots would be too difficult to decipher and would not help with interpretation of the results.

## 4 Discussion

We have proposed new loop-splitting models which facilitate local and global tests for inconsistency. Our models for loop inconsistency are the first to handle treatments symmetrically while locating inconsistency in loops rather than in nodes or treatment comparisons, and are invariant to choice of reference treatment, making the results less dependent on choice of parameterization. By choosing to assign as much variation as possible to heterogeneity rather than inconsistency, our proposed model has a simpler interpretation than alternative parameterizations and spends fewer degrees of freedom. The global model is invariant to choice of independent loops and we have shown how to identify a set of independent loops. In comparison with the existing design-by-treatment interaction approach to testing for global inconsistency in network meta-analysis, our global model uses fewer degrees of freedom and has the potential to improve power for detecting inconsistency.

When a single loop is present in the network, the potential causes of any inconsistency identified can be investigated by examining differences between the designs of trials contributing to different edges of the loop (6). Suggestions for addressing inconsistency include checking relevant study data for possible errors, using network meta-regression to explore whether study characteristics explain the inconsistency, and potentially reporting results only from inconsistency models or, in extreme cases, choosing not to synthesize the data (16). In more complex networks including multiple loops, however, we have shown that multiple different sets of independent loops produce the same global model. This makes a practical exploration of potential causes of inconsistency for any one particular set of independent loops seem less meaningful, because the data cannot always tell us where the inconsistency lies. However, in the diabetes prevention network, our inconsistency visualization plots showed that the strongest evidence for inconsistency lies in the same part of the network under each global model fitted, so the exploration of potential causes of inconsistency would be similar in each case. Approaches for testing for local inconsistency such as the side-splitting approach (7,10) would be more appropriate than our global approach if researchers have a particular interest in testing for inconsistency in one loop. The global test for inconsistency is likely to lack power in comparison with a local test.

Currently, our proposed methods are suitable for networks including pairwise and three-arm trials, but not trials including four or more arms. Our algorithm for identifying loops is generic and can identify loops within networks including an arbitrary number of treatments, but we have not found a generic method for coding the required network meta-regression covariates for trials including an arbitrary number of arms. We plan to address this limitation in further work, but note that trials including more than three parallel arms are relatively rare. Trials including many arms are often motivated by lack of evidence on useful interventions for a condition, so are less likely to be included in network meta-analyses. Our algorithm for identifying a minimal network spanning matrix involves checking all possible unions of spanning trees for the set of trial designs in the network. The number of these possible unions rises exponentially with the number of trials with more than two arms and with the number of arms (minus two) of each trial. The algorithm is completed quickly for network meta-analyses including numbers of treatments that are typical in clinical applications,but would become infeasible for networks including many multi-arm trials, or even a single trial with very many arms. An efficient algorithm would need to be developed to cope with such cases. Our methods have been proposed in the context of fitting two-stage network meta-analysis models; fitting one-stage loop inconsistency models would be more complicated and may be worth exploring.

An alternative approach to handling inconsistency is to fit random-effects inconsistency models in every network meta-analysis on the assumption that inconsistency may be present (17), rather than choosing a model following a test for inconsistency. This approach could be seen as analogous to the standard practice of fitting random effects rather than common effect models in pairwise or network meta-analysis, to allow for potential between-study heterogeneity. However, inconsistency models complicate the interpretation of observed results, so might seem unappealing as a primary analysis when there is no evidence of inconsistency. The models presented in this article assumed between-study heterogeneity to be equal across comparisons; inconsistency could potentially be estimated incorrectly if this assumption is inappropriate. Alternative models allowing heterogeneity variances to differ across treatment comparisons are available (18), but the very small numbers of studies typically informing each comparison mean that these heterogeneity variances are usually very imprecisely estimated in a frequentist framework. We would suggest using a Bayesian framework to model inconsistency while allowing unequal heterogeneity variances, in order that poorly informed heterogeneity parameters can benefit from informative priors (19); this is beyond the scope of the current article.

In networks including only pairwise trials (or in which all comparisons in multi-arm trials are also present in a pairwise trial), the number of parameters in a global loop-inconsistency model is fixed and equal to the number of comparisons informed by data. If a network includes one or more comparisons that are present only in a multi-arm trial, however, there are different options for how to parameterize a global loop-inconsistency model (8,12). We have chosen to minimize the number of inconsistency parameters in the model, meaning that variation among trials is modeled as heterogeneity wherever possible. This view differs from the approach taken by others, who preferred to maximize the number of inconsistency parameters (8,12). Our preference is based on choosing a model including fewer parameters in total, with a simpler interpretation, because between-trial heterogeneity is usually assumed equal across comparisons. In the next phase of this research, we plan to carry out a simulation study to evaluate the power of our proposed approach in comparison with other available approaches. By including inconsistency parameters only for independent loops and modeling variation as heterogeneity where possible, we have reduced the degrees of freedom required when testing for global inconsistency, in comparison with the DBTI model that allows for all design-by-treatment interactions. We recommend our proposed approach for evaluating local or global inconsistency in network meta-analysis.

## Supplementary Material

Supplementary material

## Figures and Tables

**Table 1:** Consistency model, asymmetrical and symmetrical side-splitting models, Lu-Ades inconsistency model and our proposed inconsistency model for the networks in Figure 1. Each entry shows the true mean treatment effect, on the same scale as model (1), for the given treatment contrast in studies of the given design.

Design	Consistency model^a^		
	*B* vs *A*	*C* vs *A*	*C* vs *B*
AB	*δA^B^*	—	—
AC	—	*δ^AC^*	—
BC	—	—	*δ^BC^*
ABC	*δ^AB^*	*δ^AC^*	*δ^BC^*
**Design**	**Asymmetrical model^a^: splitting side B-C**			**Symmetrical model^a^: splitting side B-C**		
	***B* vs *A***	***C* vs *A***	***C* vs *B***	***B* vs *A***	***C* vs *A***	***C* vs *B***
AB	*δA^B^*	—	—	*δ^AB^*	—	—
AC	—	*δAC*	—	—	*δ^AC^*	—
BC	—	—	*δ^BC^* + *ω*	—	—	*δ^BC^* + *ω*
ABC	*δA^B^*	*δA^C^* + *ω*	*δ^BC^* + *ω*	*δ^AB^* − *ω*/2	*δ^AC^* + *ω*/2	*δ^BC^* + *ω*
**Design**	**Inconsistency Lu-Ades model^a^: A as reference**			**Inconsistency model^a^: splitting directed loop A- > B- > C- > A**		
	***B* vs *A***	***C* vs *A***	***C* vs *B***	***B* vs *A***	***C* vs *A***	***C* vs *B***
AB	*δ^AB^*	—	—	*δ^AB^* + *ω*/3	—	—
AC	—	*δAC*	—	—	*δ^AC^* − *ω*/3	—
BC	—	—	*δ^BC^* + *ω*	—	—	*δ^BC^* + *ω*/3
ABC	*δ^AB^*	*δ^AC^*	*δ^BC^*	*δ^AB^*	*δ^AC^*	*δ^BC^*

a Note that the consistency equation *δ^BC^* = *δ^AC^* − *δ^AB^* is assumed for the *δ* parameters throughout the table and inconsistency is expressed via the *ω* parameters.

**Table 2:** Covariates for network meta-regression implementation of local loop-splitting model when splitting a single (a) three-treatment or (b) four-treatment loop excluding the reference treatment.

(a) Splitting a three-treatment loop BCD in a network with reference treatment A: covariates to be used in network meta-regression implementation			
Design^a^	Covariate for B vs A contrast	Covariate for C vs A contrast	Covariate for D vs A contrast
BC* not D	−1/2	1/2	0
CD* not B	0	−1/2	1/2
BD* not C	1/2	0	−1/2
BCD	0	0	0
**(b) Splitting a four-treatment loop BCDE in a network with reference treatment A: covariates to be used in network meta-regression implementation**			
**Design** ^a^	**Covariate for BvsA contrast**	**Covariate for CvsA contrast**	**Covariate for DvsA contrast**	**Covariate for EvsA contrast**
BC* not D or E	−1/2	1/2	0	0
CD* not B or E	0	−1/2	1/2	0
DE* not B or C	0	0	−1/2	1/2
BE* not C or D	1/2	0	0	−1/2
BD* not C or E	0	0	0	0
CE* not B or D	0	0	0	0
BCD	−1/2	0	1/2	0
CDE	0	−1/2	0	1/2
BDE	1/2	0	−1/2	0
BCE	0	1/2	0	−1/2

a Note that “BC* not D” (for example) refers to all designs including treatments B and C, and possibly other treatments, but not D.

**Table 3:** Proposed inconsistency model for splitting a four-treatment loop in an arbitrary network. Each entry shows the true mean treatment effect, on the same scale as model (1), for the given treatment contrast in studies of the given design.

Inconsistency model: splitting directed loop *B → C → D → E → B* (shown in terms of contrasts included in the loop)				
Design	*C* vs *B*	*D* vs *C*	*E* vs *D*	*E* vs *B*
BC	*δ^BC^* + *ω*/4	—	—	—
CD	—	*δ^CD^* + *ω*/4	—	—
DE	—	—	*δ^DE^* + *ω*/4	—
BE	—	—	—	*δ^BE^* − *ω*/4
BCD	*δ^BC^* + *ω*/8	*δ^CD^* + *ω*/8	—	—
CDE	—	*δ^CD^* + *ω*/8	*δ^DE^* + *ω/8*	—
BDE	—	—	*δ^DE^* + *ω/8*	*δ^BE^ − ω/8*
BCE	*δ^BC^* + *ω*/8	—	—	*δ^BE^ − ω/8*

**Table 4:** Estimated inconsistency parameters and results from testing for local or global loop-inconsistency in smoking cessation network meta-analysis.

Loop(s) split	Inconsistency parameter(s) (SE)	Wald *χ*^2^	df	*p*-value
ABC	−0.343 (0.745)	0.21	1	.65
ABD	0.055 (1.006)	0.00	1	.96
ACD	−0.343 (1.034)	0.11	1	.74
BCD	−1.443 (1.175)	1.51	1	.22
ABCD	−1.159 (1.282)	0.82	1	.37
ABDC	0.235 (0.910)	0.07	1	.80
ACBD	0.762 (1.206)	0.40	1	.53
ABD, ACD, BCD (global model 1)	−0.455 (1.101) −0.020 (1.089) −1.646 (1.366)	1.57	3	.67
ABD, ACD, ABC (global model 2)	1.191 (1.420) −1.666 (1.517) −1.646 (1.366)	1.57	3	.67
ABD, BCD, ABDC (global model 3)	−0.475 (1.558) −1.646 (1.366) 0.026 (1.453)	1.57	3	.67

**Table 5:** Estimated inconsistency parameters and results from testing for local or global loop-inconsistency in diabetes prevention network meta-analysis.

Loops split	Inconsistency parameters (SE)	Wald *χ*^2^	df	p-value
ABD, ACD, ABE, ACE, ABF,ACF, ADF (global model 1)	0.239 (0.347) -0.243 (0.256) -0.451 (0.279) 0.393 (0.295) 0.120 (0.656) 0.507 (0.662) 0.320 (0.642)	6.49	7	0.48
ABD, ACD, ADF, BDF, CDF, AEBD, AECD (global model 2)	-0.093 (0.572) 0.657 (0.583) 0.947 (1.751) -0.120 (0.656) -0.507 (0.662) 0.602 (0.372) -0.523 (0.394)	6.49	7	0.48
ABF, ACF, ADF, BDF, CDF, AFBE, AFCE (global model 3)	-0.093 (0.572) 0.657 (0.583) 0.324 (0.579) 0.239 (0.347) -0.243 (0.256) -0.602 (0.372) 0.523 (0.394)	6.49	7	0.48

**Table 6:** Estimated inconsistency parameters and results from testing for global loop-inconsistency in MANGA network meta-analysis.

Loops split	Wald *χ*^2^	df	*p*-value
ADBE, ADCE, ADE, AEFBD, ADBFGE, AEHBD, BFH, ADBI, ADCI, ADI, AEI, ADBFI, AEGI, ADBHI, AEJBD, ADBK, ADK, AEK, ADBFK, AEGK, ADBHK, AIK, ADBJK, ADBL, ADL, AEL, ADBFL, ADBHL, AIL, ADBJL, AKL (global model 1)	30.22	31	.51
CDE, EFG, EFH, CDI, CEI, CEFI, CEGI, CEHI, CEKD, CEFKD, CEGKD, CEHKD, CIKD, CEJKD, CELD, CEFLD, CEHLD, CILD, CEJLD, DKL, ADCE, ADCI, ADK, ADL, BDCE, BDCEF, BDCEH, BDCI, BDCEJ, BDK, BDL (global model 2)	30.22	31	.51
EFG, EFH, DEI, DEFI, DEGI, DEHI, DEK, DEFK, DEGK, DEHK, DIK, DEJK, DEL, DEFL, DEHL, DIL, DEJL, DKL, ADE, ADI, ADK, ADL, BDE, BDEF, BDEH, BDI, BDEJ, BDK, BDL, CDE, CDI (global model 3)	30.22	31	.51

## Data Availability

The three network meta-analysis data sets that support the findings of this study are openly available in the UCL Research Data Repository at the following links: Network meta-analysis comparing interventions for promoting smoking cessation, Network meta-analysis comparing antihypertensive drugs for prevention of diabetes, Network meta-analysis comparing efficacy of 12 anti-depressants.

## References

[1] Petropoulou M, Nikolakopoulou A, Veroniki AA (2017). Bibliographic study showed improving statistical methodology of network meta-analyses published between 1999 and 2015. J Clin Epidemiol.

[2] Higgins JPT, Whitehead A (1996). Borrowing strength from external trials in a meta-analysis. Stat Med.

[3] Lumley T (2002). Network meta-analysis for indirect treatment comparisons. Stat Med.

[4] Lu G, Ades AE (2004). Combination of direct and indirect evidence in mixed treatment comparisons. Stat Med.

[5] Caldwell DM, Ades AE, Higgins JPT (2005). Simultaneous comparison of multiple treatments: combining direct and indirect evidence. Brit Med J.

[6] Salanti G, Higgins JPT, Ades AE, Ioannidis JPA (2008). Evaluation of networks of randomized trials. Stat Methods Med Res.

[7] Dias S, Welton NJ, Caldwell DM, Ades AE (2010). Checking consistency in mixed treatment comparison meta-analysis. Stat Med.

[8] Lu G, Ades AE (2006). Assessing evidence inconsistency in mixed treatment comparisons. J Am Stat Assoc.

[9] Higgins JPT, Jackson D, Barrett JK, Lu G, Ades AE, White IR (2012). Consistency and inconsistency in network meta-analysis: concepts and models for multi-arm studies. Res Synth Methods.

[10] White IR (2015). Network meta-analysis. Stata J.

[11] White IR, Barrett JK, Jackson D, Higgins JPT (2012). Consistency and inconsistency in network meta-analysis: model estimation using multivariate meta-regression. Res Synth Methods.

[12] van Valkenhoef G, Tervonen T, de Brock B, Hillege H (2012). Algorithmic parameterization of mixed treatment comparisons. Stat Comput.

[13] Hasselblad V (1998). Meta-analysis of multi-treatment studies. Med Decis Making.

[14] Elliott WJ, Meyer PM (2007). Incident diabetes in clinical trials of antihypertensive drugs: a network meta-analysis. Lancet.

[15] Cipriani A, Furukawa TA, Salanti G (2009). Comparative efficacy and acceptability of 12 new-generation antidepressants: a multiple-treatments meta-analysis. Lancet.

[16] Cipriani A, Higgins JPT, Geddes JR, Salanti G (2013). Conceptual and technical challenges in network meta-analysis. Ann Intern Med.

[17] Jackson D, Barrett JK, Rice S, White IR, Higgins JPT (2014). A design-by-treatment interaction model for network meta-analysis with random inconsistency effects. Stat Med.

[18] Lu G, Ades AE (2009). Modeling between-trial variance structure in mixed treatment comparisons. Biostatistics.

[19] Turner RM, Dominguez-Islas CP, Jackson D, Rhodes KM, White IR (2019). Incorporating external evidence on between-trial heterogeneity in network meta-analysis. Stat Med.

