# Supplementary material for: A new approach to evaluating loop inconsistency in network meta-analysis
Source: Stat Med. Author manuscript; Available in PMC 2023 Dec 6. (PMC7615349; doi:10.1002/sim.9872)
Supplement: Supplementary material [file EMS190515-supplement-Supplementary_material.pdf]

Network meta-regression implementation of local loop-splitting model

Table S1 Covariates for network meta-regression implementation of local loop-splitting model when splitting a single five-treatment loop excluding the reference treatment.

Splitting a five-treatment loop BCDEF in a network with reference treatment A: covariates to be used in network meta-regression implementation.					
Design	Covariate for B vs. A contrast	Covariate for C vs. A contrast	Covariate for D vs. A contrast	Covariate for E vs. A contrast	Covariate for F vs. A contrast
BC* not D,E,F	-1/2	1/2	0	0	0
CD* not B,E,F	0	-1/2	1/2	0	0
DE* not B,C,F	0	0	-1/2	1/2	0
EF* not B,C,D	0	0	0	-1/2	1/2
BF* not C,D,E	1/2	0	0	0	-1/2
BD* not C,E,F	0	0	0	0	0
BE* not C,D,F	0	0	0	0	0
CE* not B,D,F	0	0	0	0	0
CF* not B,D,E	0	0	0	0	0
DF* not B,C,E	0	0	0	0	0
BCD	-1/2	0	1/2	0	0
BCE	-1/2	1/2	0	0	0
BCF	0	1/2	0	0	-1/2
BDE	0	0	-1/2	1/2	0
BDF	1/2	0	0	0	-1/2
BEF	1/2	0	0	-1/2	0
CDE	0	-1/2	0	1/2	0
CDF	0	-1/2	1/2	0	0
CEF	0	0	0	-1/2	1/2
DEF	0	0	-1/2	0	1/2

For loops including the reference treatment, the covariates used in the network meta-regression implementation are defined as in the examples shown in Tables S2 and S3. We note the special case of a three-arm trial for which two treatments are included in a loop, for example an ABD trial informing an ABC loop. The trial is internally consistent and therefore if the AB comparison is inconsistent with the rest of the network, the BD and AD comparisons must also be inconsistent with the rest of the network. The practical implication of this is that if we assign an inconsistency term of $\omega/3$ to AB (for a three-treatment loop), we should also assign inconsistency terms of $\omega/6$ to each of AD and DB (in order that they sum to $\omega/3$). For loop ABC including the reference treatment, this is achieved by assigning a non-zero covariate value to the D vs. A contrast, as shown in Table S2.

Table S2 Covariates for network meta-regression implementation of local loop-splitting model when splitting a single three-treatment or four-treatment loop including the reference treatment.

Splitting a three-treatment loop ABC in a network with reference treatment A: covariates to be used in network meta-regression implementation.			
Design	Covariate for B vs. A contrast	Covariate for C vs. A contrast	Covariate for D vs. A contrast
AB	1	0	0
BC	-1/2	1/2	0
AC	0	-1	0
ABC	0	0	0
ABD	1	0	1/2

	Splitting a four-treatment loop ABCD in a network with reference treatment A: covariates to be used in network meta-regression implementation.			
Design	Covariate for B vs. A contrast	Covariate for C vs. A contrast	Covariate for D vs. A contrast	Covariate for E vs. A contrast
AB	1	0	0	0
BC	-1/2	1/2	0	0
CD	0	-1/2	1/2	0
AD	0	0	-1	0
AC	0	0	0	0
BD	0	0	0	0
ABC	1/2	1	0	0
BCD	-1/2	0	1/2	0
ACD	0	-1	-1/2	0
ABD	1/2	0	-1/2	0
ABE	1	0	0	1/2

Table S3 Covariates for network meta-regression implementation of local loop-splitting model when splitting a single five-treatment loop including the reference treatment.

Splitting a five-treatment loop ABCDE in a network with reference treatment A: covariates to be used in network meta-regression implementation.					
Design	Covariate for B vs. A contrast	Covariate for C vs. A contrast	Covariate for D vs. A contrast	Covariate for E vs. A contrast	Covariate for F vs. A contrast
AB	1	0	0	0	0
BC	-1/2	1/2	0	0	0
CD	0	-1/2	1/2	0	0
DE	0	0	-1/2	1/2	0
AE	0	0	0	-1	0
AC	0	0	0	0	0
AD	0	0	0	0	0
BD	0	0	0	0	0
BE	0	0	0	0	0
CE	0	0	0	0	0
ABC	1/2	1	0	0	0
ABD	1	0	1/2	0	0
ABE	1/2	0	0	-1/2	0
ACD	0	-1/2	1/2	0	0
ACE	0	-1/2	0	-1	0
ADE	0	0	-1	-1/2	0
BCD	-1/2	0	1/2	0	0
BCE	-1/2	1/2	0	0	0
BDE	0	0	-1/2	1/2	0
CDE	0	-1/2	0	1/2	0
ABF	1	0	0	0	1/2

Algorithm for identifying a model parameterisation that minimises the number of loops

The code below assumes that the initial network meta-analysis data set is stored in “long” form, containing one row per treatment per study, with the following variables: `study`, `treat`, `event`, `total`. The data set is then converted to an “augmented” form using the `network setup` command (1).

Stata do-file (with embedded Mata code) for identifying a minimal network spanning matrix, then finding a set of independent loops created by this matrix

```
* Set up network and create treatment indicators
egen arms=count(treat), by(study)
qui summ arms
local maxarms=r(max)
network setup event total, studyvar(study) trtvar(treat)

forvalues i=1/`maxarms' {
       gen treat`i'=word(_design,`i')
       }
preserve
keep study treat*
reshape long treat, i(study) j(arm)
levelsof treat, local(trtlevels) clean
restore
foreach treat in `trtlevels' {
       gen includes`treat'=strpos(_design,"`treat'")
       replace includes`treat'=1 if includes`treat'>=1
       }

mata
includes=st_data(.,("includes*"))
trials=rows(includes)
trts=cols(includes)
// Create network adjacency matrix based on pairwise trials
adjacency_pw=J(trts,trts,0)
       for (i=1;i<=trials;++i) {
              for (j=1;j<=trts;++j) {
                     for (k=1;k<=trts;++k) {
                            if (includes[i,j]==1 & includes[i,k]==1 &
sum(includes[i,.])==2) adjacency_pw[j,k]=1
                            if (j==k) adjacency_pw[j,k]=0
                     }
              }
       }

// Create adjacency and indirect connections matrices for each trial, to generate
spanned/unspanned indicator
spanned=J(trials,1,.)
for (i=1;i<=trials;i++) {
       adj_part_i=select(adjacency_pw,includes[i,.])
       adji=select(adj_part_i,includes[i,.]')
       st_matrix("adj"+strofreal(i),adji)
       indirecti=adji
       for (j=2;j<trts;++j) {
              indirecti=indirecti+adji*indirecti
       }
       indirecti=indirecti:>0
       trtsi=rows(indirecti)
       spanned[i]=(indirecti==J(trtsi,trtsi,1))
       st_matrix("indirect"+strofreal(i),indirecti)
}

unspanned=J(0,1,.)
for (i=1;i<=trials;i++) {
       unspanned=unspanned\(i*(spanned[i]==0))
```

```
}
unspanned=select(unspanned,unspanned[.]:>0)

// Generate all network adjacency matrices which span every trial and choose one
with fewest edges
minimal_adjacency=spanNetwork(adjacency_pw,unspanned)


// Extract set of edges from network adjacency matrix
edges=J(0,3,.)
trtsk=rows(minimal_adjacency)
for (i=1;i<=trtsk;i++) {
	for (j=1;j<i;j++) {
		edges=edges\(j,i,(minimal_adjacency[i,j]==1))
	}
}
edges=select(edges,edges[.,3]:==1)
edges=select(edges,(1,1,0))

Tree=edges[1,.]
root=Tree[1]


// Add edges to tree if they are connected by one vertex, rerun loop until no more
edges are added
do {
	edgeAdded=0
	for (i=1;i<=rows(edges);i++) {
		if
(vertexInTree(edges[i,1],Tree)&!vertexInTree(edges[i,2],Tree)&!edgeInTree(edges[i,.
],Tree)) {
			Tree=Tree\edges[i,.]
			edgeAdded=1
		}
		if
(!vertexInTree(edges[i,1],Tree)&vertexInTree(edges[i,2],Tree)&!edgeInTree(edges[i,.
],Tree)) {
			Tree=Tree\(edges[i,2],edges[i,1])
			edgeAdded=1
		}
	}
} while (edgeAdded==1)


// Find loops created by edges for which both vertices are included in spanning
tree
for (i=1;i<=rows(edges);i++) {
	vertex1=edges[i,1]
	vertex2=edges[i,2]
	if
(vertexInTree(vertex1,Tree)&vertexInTree(vertex2,Tree)&!edgeInTree(edges[i,.],Tree)
) {
		findLoop(vertex1,vertex2,Tree)
	}
}

end
```

Stata do-file (with embedded Mata code) defining spanNetwork function

```
version 15
mata:

real matrix spanNetwork(real matrix arg1,real colvector arg2) {

	real scalar k, adjmatrixtotal, first_trial
	real colvector unspanned, other_trials
```

```
	real matrix adjacency, adjmatrixarray, adjacency_minimal, adjacencyk, 
adjacency_completed

	adjacency=arg1
	unspanned=arg2

	// Return current adjacency matrix if there are no remaining unspanned 
trials
	if (rows(unspanned)==0) {
		return(adjacency)
	}

	// Choose one trial to span first, and store set of other unspanned trials
	first_trial=unspanned[1]
	if (rows(unspanned)>1) {
		other_trials=unspanned[2..rows(unspanned)]
	}
	else other_trials=J(0,0,.)

	// Apply spanTrial() to first trial, and generate set of updated network 
adjacency matrices which span this trial
	printf("Calling spanTrial for trial "+strofreal(first_trial)+"\n")
	adjmatrixarray=spanTrial(first_trial,adjacency)
	adjmatrixtotal=asarray_elements(adjmatrixarray)

	adjacency_minimal=J(0,0,.)

	// Loop through set of updated network adjacency matrices
	for(k=1;k<=adjmatrixtotal;k++) {
		adjacencyk=asarray(adjmatrixarray,k)
		// If there are no remaining unspanned trials, save current adjacency 
matrix as a completed network adjacency matrix
		if (rows(other_trials)==0) {
			adjacency_completed=adjacencyk
		}
		// If there are remaining unspanned trials, apply spanNetwork() and 
save returned value as a completed network adjacency matrix
		else {
			printf("Calling spanNetwork, iteration "+strofreal(k)+"\n")
			adjacency_completed=spanNetwork(adjacencyk,other_trials)
		}
		printf("Completed adjacency matrix:"+"\n")
		adjacency_completed

		// Choose completed network adjacency matrix with the fewest edges
		if 
((sum(adjacency_completed:==1)<sum(adjacency_minimal:==1))|adjacency_minimal==J(0,0
,.)) {
			adjacency_minimal=adjacency_completed
		}
	}

return(adjacency_minimal)

}

mata mosave spanNetwork()
end
```

Stata do-file (with embedded Mata code) defining spanTrial function

```
version 15
mata:

function spanTrial(real scalar arg1,real matrix arg2) {

	real scalar k, trtsk, j, i, trtsk_i, spannedk_i, newmatrixcount, l, 
adjmatrixcount
	real rowvector treatlistk_i
```

```
	real matrix adjacency, includes, A, adj_part_k, adjk, indirectk, coords, 
coords0, adjk_i, indirectk_i, adjacency_i, newarray_spanning, adjacency_l

	k=arg1
	adjacency=arg2
	includes=st_data(.,("includes*"))
	A=asarray_create("real",1)

	// Create adjacency and indirect connections matrix for this trial
	adj_part_k=select(adjacency,includes[k,.])
	adjk=select(adj_part_k,includes[k,.]')
	trtsk=rows(adjk)
	indirectk=adjk
	for (j=2;j<trtsk;j++) {
		indirectk=indirectk+adjk*indirectk
	}
	indirectk=indirectk:>0

	// Identify treatment pairs with zero entries in trial's indirect 
connections matrix
	coords=J(0,3,.)
	for (i=1;i<=trtsk;i++) {
		for (j=1;j<i;j++) {
			coords=coords\(i,j,(indirectk[i,j]==1))
		}
	}
	coords0=select(coords,coords[.,3]:==0)
	coords0=select(coords0,(1,1,0))

	// Return current network adjacency matrix if there are no treatment pairs 
with zero entries (i.e. trial is already spanned)
	if (rows(coords0)==0) {
	printf("Trial "+strofreal(k)+" is already spanned\n")
	asarray(A,1,adjacency)
	return(A)
	}

	// Loop through treatment pairs with zero entries
	for (i=1;i<=rows(coords0);i++) {
		// Add edge corresponding to current treatment pair to trial's 
adjacency matrix
		adjk_i=adjk
		adjk_i[coords0[i,1],coords0[i,2]]=1
		adjk_i[coords0[i,2],coords0[i,1]]=1
		trtsk_i=rows(adjk_i)
		// Update trial's indirect connections matrix
		indirectk_i=adjk_i
		for(j=2;j<trtsk_i;j++) {
			indirectk_i=indirectk_i+adjk_i*indirectk_i
		}
		indirectk_i=indirectk_i:>0
		// Create indicator of whether trial k is now spanned
		spannedk_i=(indirectk_i==J(trtsk_i,trtsk_i,1))
		// Map treatment labels in trial to treatment labels for whole network
		treatlistk_i=J(1,cols(includes),.)
		for (j=1;j<=cols(includes);j++) {
			treatlistk_i[1,j]=j*(includes[k,j]==1)
		}
		treatlistk_i=select(treatlistk_i,treatlistk_i:>0)
		// Update network adjacency matrix
		adjacency_i=adjacency
		adjacency_i[treatlistk_i[coords0[i,1]],treatlistk_i[coords0[i,2]]]=1
		adjacency_i[treatlistk_i[coords0[i,2]],treatlistk_i[coords0[i,1]]]=1
		// If trial k is spanned, add updated network adjacency matrix to 
array A
		if (spannedk_i==1) {
			printf("Updated adjacency matrix for trial "+strofreal(k))
			adjk_i
			printf("Updated adjacency matrix for network")
			adjacency_i
```

```
                    adjmatrixcount=asarray_elements(A)
                    asarray(A,adjmatrixcount+1,adjacency_i)
                }
                // If trial k is not spanned, apply spanTrial() to updated network
adjacency matrix and add returned values to array A
                else {
                    printf("Calling spanTrial for trial "+strofreal(k)+", iteration
"+strofreal(i) + "\n")
                    newarray_spanning=spanTrial(k,adjacency_i)
                    newmatrixcount=asarray_elements(newarray_spanning)
                    for (l=1;l<=newmatrixcount;l++) {
                        adjacency_l=asarray(newarray_spanning,l)
                        adjmatrixcount=asarray_elements(A)
                        asarray(A,adjmatrixcount+1,adjacency_l)
                    }

                }
        }
        return(A)
}

mata mosave spanTrial()
end
```

Stata do-file (with embedded Mata code) defining the following functions: edgeInTree, vertexInTree, findAncestry, findLCA (lowest common ancestor), findLoop

```
version 15
mata:

// Define function to check whether edge is included in tree
real scalar edgeInTree(real rowvector arg1,real matrix arg2) {
        edge=arg1
        Tree=arg2
        InTree=J(rows(Tree),1,.)
        for (i=1;i<=rows(Tree);i++) {
                if (Tree[i,.]==edge|(Tree[i,1]==edge[2]&Tree[i,2]==edge[1])) {
                InTree[i]=1
                }
        }
return(sum(InTree))
}
mata mosave edgeInTree()

// Define function to check whether vertex is included in tree
real scalar vertexInTree(real scalar arg1,real matrix arg2) {
        vertex=arg1
        Tree=arg2
        InTree=J(rows(Tree),2,.)
        for (i=1;i<=rows(Tree);i++) {
                for (j=1;j<=2;j++) {
                        if (Tree[i,j]==vertex) {
                                InTree[i,j]=1
                        }
                }
        }
indicator=(sum(InTree)>=1)
return(indicator)
}
mata mosave vertexInTree()

// Define function to find ancestry of a vertex from the root of a tree
real rowvector findAncestry(real scalar arg1,real matrix arg2) {
        vertex=arg1
        Tree=arg2
        ancestry=J(1,1,vertex)
        do {
                for (i=1;i<=rows(Tree);i++) {
```

```
                    if (Tree[i,2]==ancestry[1]) {
                        ancestry=(Tree[i,1]),ancestry
                    }
                }
        } while (ancestry[1]!=1)
return(ancestry)
}
mata mosave findAncestry()

// Define function to find lowest common ancestor of two vertices
real scalar findLCA(real scalar arg1,real scalar arg2,real matrix arg3) {
        vertex1=arg1
        vertex2=arg2
        Tree=arg3
        ancestry1=findAncestry(vertex1,Tree)
        ancestry2=findAncestry(vertex2,Tree)
        for(i=1;i<=length(ancestry1)&i<=length(ancestry2);i++) {
                if (ancestry1[i]!=ancestry2[i]) {
                        LCA=ancestry1[i-1]
                        break
                }
        }
if (length(LCA)==0) _error("one vertex is an ancestor of the other")
return(LCA)
}
mata mosave findLCA()

// Define function to find loop created by joining two vertices already included in
spanning tree
real rowvector findLoop(real scalar arg1,real scalar arg2,real matrix arg3) {
        vertex1=arg1
        vertex2=arg2
        Tree=arg3
        ancestry1=findAncestry(vertex1,Tree)
        ancestry2=findAncestry(vertex2,Tree)
        LCA=findLCA(vertex1,vertex2,Tree)
        loopPart1=J(1,0,.)
        for (j=1;j<=length(ancestry1);j++) {
                if (ancestry1[j]==LCA|length(loopPart1)!=0) {
                        loopPart1=loopPart1,ancestry1[j]
                }
        }
        loopPart2=J(1,0,.)
        for (j=1;j<=length(ancestry2);j++) {
                if (ancestry2[j]==LCA|length(loopPart2)!=0) {
                        loopPart2=loopPart2,ancestry2[j]
                }
        }
        loopPart2=loopPart2[2..length(loopPart2)]
        loop=loopPart1
        for (j=length(loopPart2);j>=1;j--) {
                loop=loop,loopPart2[j]
        }
return(loop)
}
mata mosave findLoop()


end
```

Stata ado-file defining the loopsplitting function to split a single loop

```
program define network_loopsplitting

* PARSING
syntax namelist(min=3), [BSCOVariance(passthru) fixed options]
local loopall `namelist'
local loopall_tokenize `loopall'
```

```
local loopsize=wordcount("`loopall'")
local loopname ""
forvalues i=1/`loopsize' {
	gettoken loop`i' loopall_tokenize : loopall_tokenize
	local loopname = "`loopname'" + "`loop`i''"
}

// LOAD SAVED NETWORK PARAMETERS
foreach thing in `_dta[network_allthings]' {
	local `thing' : char _dta[network_`thing']
}

local trtlist `ref' `trtlistnoref'

// check args are known treatments
local diff : list loopall - trtlist
if !mi("`diff'") {
	di as error "Unknown treatment(s): `diff'"
	exit 198
}

// check trials include no more than 3 treatments
local designlength=strlen(_design)
if `designlength'>5 {
	di as error "Not applicable to trials including 4 or more treatments"
	exit 198
}


// START
preserve
cap network convert augmented
foreach dropvar of local metavars {
	cap drop `dropvar'
}
local metavars

// define contrasts, with separate cases for trials sharing one edge with loop or
two edges with loop
foreach trt of local trtlistnoref {
	gen _inco`trt' = 0
	local metavars `metavars' _inco`trt'
}
tempvar thisdesign_oneedge thisdesign_twoedges
gen byte `thisdesign_oneedge' = 0
gen byte `thisdesign_twoedges' = 0

forvalues i=1/`loopsize' {
	local iplus1 = 1 + mod(`i', `loopsize')
	local iplus2 = 1 + mod(`i'+1, `loopsize')

	local trt1 `loop`i''
	local trt2 `loop`iplus1''
	local trt3 `loop`iplus2''

	qui replace `thisdesign_oneedge' = strpos(" " + _design + " ", " `trt1' ") &
strpos(" " + _design + " ", " `trt2' ") & !strpos(" " + _design + " ", " `trt3' ")
	qui replace `thisdesign_oneedge' = 0 if `thisdesign_twoedges'!=0
	qui replace `thisdesign_twoedges' = strpos(" " + _design + " ", " `trt1' ")
& strpos(" " + _design + " ", " `trt2' ") & strpos(" " + _design + " ", " `trt3' ")

	local loopnot : list trtlist - loopall
	if "`trt1'"=="`ref'" {
		qui replace _inco`trt2' = 1 if `thisdesign_oneedge'
		qui replace _inco`trt2' = 1/2 if `thisdesign_twoedges'&`loopsize'>=4
		qui replace _inco`trt3' = 1 if `thisdesign_twoedges'&`loopsize'>=4
		foreach trt of local loopnot {
			qui replace _inco`trt' = 1/2 if `thisdesign_oneedge' & strpos("
" + _design + " ", " `trt' ")
		}
```

```
        }
        else if "`trt2'"=="`ref'" {
                qui replace _inco`trt1' = -1 if `thisdesign_oneedge'
                qui replace _inco`trt1' = -1/2 if `thisdesign_twoedges'&`loopsize'>=4
                qui replace _inco`trt3' = 1/2 if `thisdesign_twoedges'&`loopsize'>=4
                foreach trt of local loopnot {
                        qui replace _inco`trt' = -1/2 if `thisdesign_oneedge' &
strpos(" " + _design + " ", " `trt' ")
                }
        }
        else if "`trt3'"=="`ref'" {
                qui replace _inco`trt1' = -1/2 if `thisdesign_oneedge'
                qui replace _inco`trt2' = 1/2 if `thisdesign_oneedge'
                qui replace _inco`trt1' = -1 if `thisdesign_twoedges'&`loopsize'>=4
                qui replace _inco`trt2' = -1/2 if `thisdesign_twoedges'&`loopsize'>=4
        }
        else {
                qui replace _inco`trt1' = -1/2 if `thisdesign_oneedge'
                qui replace _inco`trt2' =  1/2 if `thisdesign_oneedge'
                qui replace _inco`trt1' = -1/2 if `thisdesign_twoedges'&`loopsize'>=4
                qui replace _inco`trt3' =  1/2 if `thisdesign_twoedges'&`loopsize'>=4
        }
}
tabstat _inco*, by(_des) nototal

// run model
gen _trtdiffzero = 0
gen _trtdiffone  = 1
local metavars `metavars' _trtdiffzero _trtdiffone
local first 1
foreach trt of local trtlistnoref {
        gen _trtdiff`trt' = `first'
        rename _inco`trt' _inco`trt'_`loopname'
        local metavars `metavars' _trtdiff`trt' _inco`trt'_`loopname'
        local eq`trt' `y'_`trt':
        foreach trt2 of local trtlistnoref {
                if `first' local eq`trt' `eq`trt'' _trtdiff`trt2'
                else if "`trt2'"=="`trt'" local eq`trt' `eq`trt'' _trtdiffone
                else if "`trt2'"!="`trt'" local eq`trt' `eq`trt'' _trtdiffzero
        }
        local eq`trt' `eq`trt'' _inco`trt'_`loopname'
        if `first' local eqs `eq`trt''
        if `first' local trt1 `trt'
        else local eqs `eqs', `eq`trt''
        local first 0
}

if mi("`bscovariance'`fixed'") local bscovariance bscovariance(exch 0.5)
global F9 mvmeta `y' `S', `bscovariance' `fixed' eq( `eqs' ) ///
        commonparm noconst network(sidesplit) suppress(uv mm)
di as input "Command is: $F9"
$F9
char _dta[network_metavars] `metavars'
di as text ""
di as text "Test for inconsistency in loop `loopname':"
lincom `loopsize'*_inco`trt1'_`loopname'
restore, not

end
```

Example Stata code for splitting loops in the smoking cessation data set

Fit a loop-inconsistency model splitting the ABC loop

```
network loopsplitting A B C
```

Fit a global loop-inconsistency model splitting the ABD, ACD and ABD loops simultaneously

```
network loopsplitting A B D
rename (_inco*) (inco*)
network loopsplitting A C D
rename (_inco*) (inco*)
network loopsplitting B C D
rename (_inco*) (inco*)

mvmeta _y _S, bscov(exch 0.5) eq(_y_B: _trtdiffB _trtdiffC _trtdiffD incoB_*, _y_C:
_trtdiffzero _trtdiffone  _trtdiffzero incoC_*, _y_D: _trtdiffzero _trtdiffzero
_trtdiffone  incoD_*) commonparm noconst network(sidesplit) suppress(uv mm)

lincom 3*incoB_ABD
lincom 3*incoB_ACD
lincom 3*incoB_BCD

testparm inco*
```

References

1. White IR. Network meta-analysis. Stata Journal. 2015;15:1-34.
